# Design load analysis for electrification of a 55-kW agricultural tractor based on workload

**DOI:** 10.1038/s41598-026-45430-3

**Published:** 2026-06-05

**Authors:** Seung-Min Baek, Hyeon-Ho Jeon, Wan-Soo Kim, Yeon-Soo Kim, Yong-Joo Kim

**Affiliations:** 1 Eco-friendly Hydrogen Electric Tractor & Agricultural Machinery Institute, Chungnam National University, Daejeon, 34134 Republic of Korea; 2 Department of Smart Agriculture Systems, Chungnam National University, Daejeon, 34134 Republic of Korea; 3 Department of Smart Bio-Industrial Mechanical Engineering, Kyungpook National University, Daegu, 41566 Republic of Korea; 4 Upland Field Machinery Research Center, Kyungpook National University, Daegu, 41566 Republic of Korea; 5 Department of Bio-Industrial Machinery Engineering, Pusan National University, Miryang, 50463 Republic of Korea; 6 Department of Smart Agriculture Systems Machinery Engineering, Chungnam National University, Daejeon, 34134 Republic of Korea

**Keywords:** Engineering, Mechanical engineering

## Abstract

This study aimed to determine and analyze the design loads required for the electrification of a 55‑kW agricultural tractor through field experiments. A measurement system was installed to record data from the engine, driving axles, power take‑off (PTO), and hydraulic pump during plow tillage, rotary tillage, and driving operation in a silt loam paddy field. The study specifically focused on power requirement analysis, load duration distribution (LDD), and rainflow counting (RFC)–based load spectrum generation for durability assessment of the electric tractor powertrain. Plow tillage imposed the highest loads, with total power peaking at 52.6 kW (95% of rated power) dominated by axle torque, while rotary tillage was PTO‑driven and driving operation showed low average loads with intermittent traction peaks. Compared with a previously studied 78‑kW tractor, the 55‑kW tractor exhibited lower overall power requirement and a more traction‑balanced distribution, while hydraulic requirements remained minimal. LDD and RFC analyses revealed that a few load cases and low‑amplitude cycles dominate the operating profile, and critical high‑load cycles occur primarily during tillage. These findings provide essential design data for electric powertrain components and establish a systematic measurement‑to‑spectrum methodology for deriving design loads for agricultural machinery, supporting the future development of utility electric tractors and durability‑driven powertrain design.

## Introduction

A tractor is a versatile agricultural vehicle that serves as a primary power source for diverse field operations. Its three major functions include: (1) towing implements for plowing and trailer transport, (2) providing rotational power via the power take-off (PTO) for operations such as rotary tillage, and (3) supplying hydraulic power for tasks like bale wrapping and loader operation^1–3^. Accurate prediction of power requirement and dynamic load characteristics is essential for designing tractor power sources and transmission systems^4–6^. Tractor power requirements are highly dependent on mechanical and hydraulic loads, which vary with torque and rotational speed under different field conditions. Because these functions often operate simultaneously under variable soil and field environments, the resulting power demand is highly dynamic and operation dependent^7–9^. Therefore, workload-based quantification of subsystem-level power distribution is critical for reliable powertrain design.

Numerous studies have measured and analyzed dynamic tractor loads to improve durability and design optimization^10–12^. Field-measured data have been processed into load spectra, equivalent loads, damage sums, and load severity indices^13–15^. Kim et al.^16^ evaluated the fatigue life of spiral bevel gears using rainflow cycle counting (RFC), the Smith–Watson–Topper model, and S–N curves derived from field-measured torque data, revealing that fatigue life varies drastically among operations, from hundreds of hours under subsoiler tillage to hundreds of thousands under rotary tillage. Kim et al.^17^ also assessed gear durability in a 78-kW tractor using equivalent torque and speed analysis and accelerated life testing (ALT). Recent studies have introduced wireless PTO torque measurement systems and embedded display and warning systems for velocity ratio and wheel slip, enabling real-time monitoring and workload-based safety improvements during tillage operations^18,19^.

In parallel with these developments, agricultural machinery electrification has gained increasing attention due to tightening emission regulations and advances in electric motor and battery technologies. Unlike internal combustion engine (ICE) tractors, electric powertrains exhibit fundamentally different torque characteristics, including high instantaneous torque capability and rapid transient response^20–22^. While these features can enhance operational performance, they may also alter drivetrain load patterns and fatigue behavior. Consequently, accurate workload characterization becomes even more important when transitioning from ICE-based systems to electric powertrains.

Despite these advances, comprehensive workload data for tractor electrification remain limited. Most previous studies have focused on ICE tractors and fatigue life evaluation, rather than providing design loads for electric tractors^23,24^. In Korea, utility tractors of 55-kW are widely used and are potential candidates for full electrification, yet their design is constrained by motor sizing and battery capacity. As battery and motor efficiency improve, accurate workload characterization and design load analysis are critical for motor, battery, and gear reducer selection^25–28^. Providing such data benefits manufacturers through component design and life prediction, and farmers by enabling reliable and efficient electric tractors for diverse operations^29,30^. However, few studies have systematically quantified subsystem-level power distribution and torque spectra required for motor-driven architectures in this power class. In particular, the 55-kW utility segment represents a practical and commercially relevant electrification target due to its moderate power demand and broad operational usage. Establishing representative design loads for this class is therefore essential for balancing motor capacity, battery energy storage, gearbox durability, and hydraulic system requirements within electrified configurations.

To address this gap, this study aimed to determine and analyze the design loads of a 55-kW agricultural tractor to support electrification. Field experiments were conducted during plow tillage, rotary tillage, and driving operations in a silt-loam paddy field. Torque and speed data from the engine, driving axles, PTO, and hydraulic pump were measured and post-processed through power requirement analysis, load duration distribution (LDD), and RFC to generate load spectra. Such analysis is essential for tractor electrification because power requirement analysis identifies the actual load levels needed for accurate motor and battery sizing, LDD captures dominant load magnitudes, exposure durations, and operating speeds for gear and bearing evaluation, and RFC-based load spectra reveal cyclic load characteristics and critical high-load events affecting fatigue life of shafts, housings, and carriers.

By integrating subsystem-level power analysis with LDD and RFC-based load spectrum generation, this study establishes a direct linkage between field-measured workload characteristics and practical drivetrain design requirements. By combining these analyses, the design loads required for e-powertrain components were quantified and evaluated, providing practical data for the electrification of 55-kW tractors. The resulting workload data and methodology establish a systematic framework for deriving tractor design loads, supporting durability-driven component design, fatigue life prediction, and the development of utility electric tractor powertrains.

## Methods

### Agricultural tractor

**Fig. 1:**
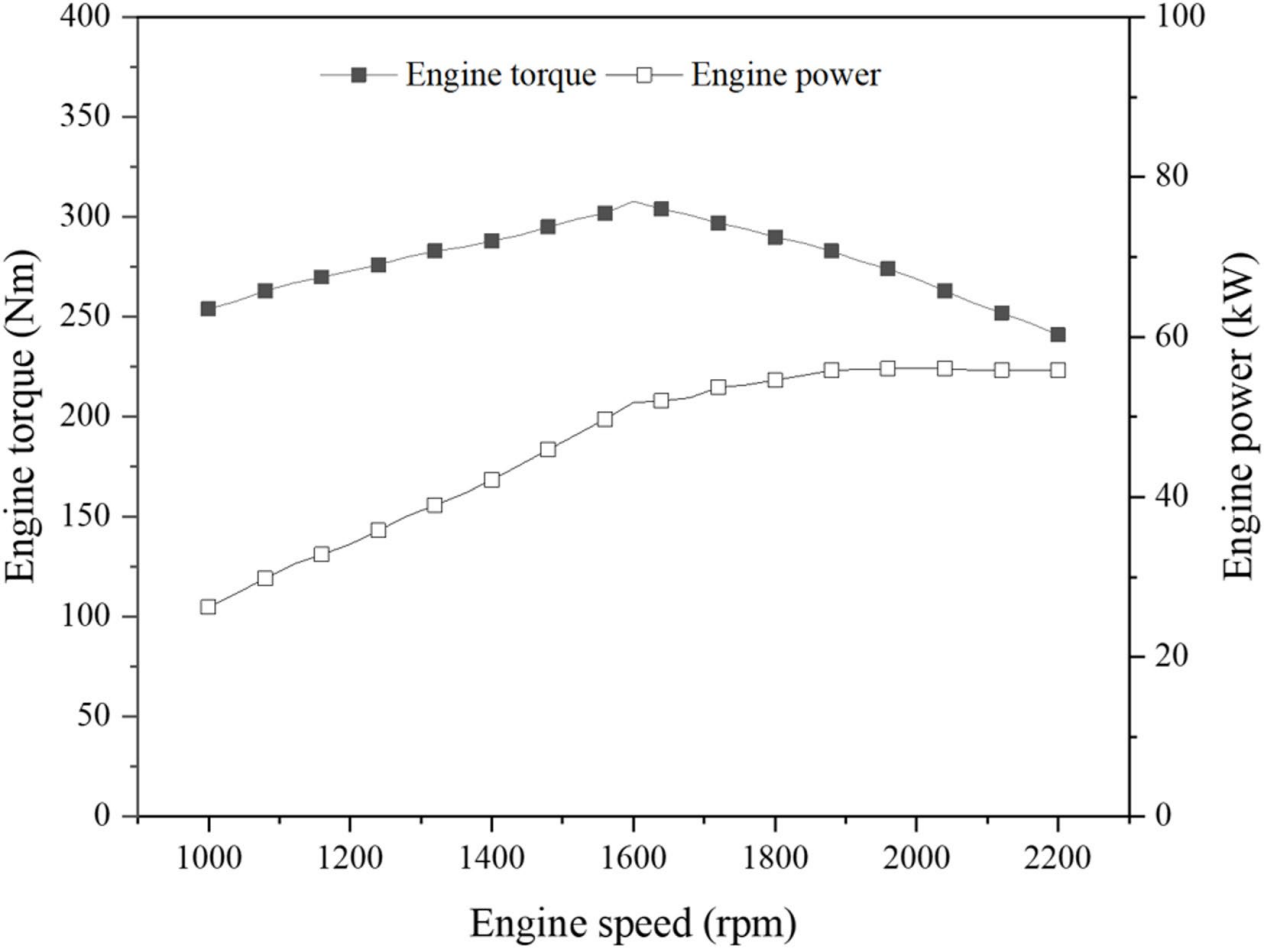
Engine T–N curve of the 55-kW agricultural tractor used in this study.

A 55-kW agricultural tractor (Agricultural Tractor for Load Measurement, NEFC-2025-06-306466) was used for the load measurements. The tractor is powered by a 55.3-kW diesel engine rated at 2200 rpm, with a peak torque of 300 Nm at 1600 rpm. The engine torque–speed (T–N) characteristics are presented in Fig. 1. The tractor dimensions are 3920 × 1940 × 2710 mm, with an empty weight of 2865 kgf and a static load distribution of 42.4:57.6 (front:rear). The powertrain consists of a mechanical transmission with a power-shuttle reverse system, providing 24 forward and 24 reverse gears. Engine power is distributed to the driving axles, PTO, and hydraulic system. Traction power is transmitted through the transmission to the front and rear axles, while PTO power is delivered directly from the engine. Hydraulic power for the three-point hitch and steering system is supplied by the main and sub hydraulic pumps. The detailed specifications of the tractor are summarized in Table 1.

**Table 1:** Specifications of the 55-kW agricultural tractor used in this study.

Item		Value
Model		TX76
Manufacturer		TYM Co., Ltd., Korea
Length $\times$ Width $\times$ Height (mm)		3920 $\times$ 1940 $\times$ 2710
Empty weight (kgf)		2865
Static load distribution (front:rear, %)		42.4:57.6
Engine	Rated power (kW)	55.3 @2200 rpm
Transmission	Type	Power shuttle
	Gear stage	Forward 24/Reverse 24
Hydraulic pump	Main	20 cc/rev
	Sub	10 cc/rev
Tire	Front	11.2–24
	Rear	16.9–30

### Load measurement system

The tractor was instrumented to measure torque, rotational speed, hydraulic pressure, and flow rate at the major power transmission points, including the engine, driving axles, PTO shaft, and hydraulic pumps. Engine torque and rotational speed were acquired through the controller area network (CAN) interface. Axle and PTO torques were measured using telemetry-based torque sensors installed on the respective shafts. The rotational speed of the driving axles was measured using proximity sensors installed adjacent to a 100-tooth trigger gear mounted on each axle shaft. The passage of each gear tooth generated a pulse signal, and the shaft rotational speed was calculated from the pulse frequency. Hydraulic pressure was measured at the outlet of the main and sub pumps, and volumetric flow rate was measured downstream of each pump. Hydraulic power was calculated from the measured pressure and flow rate, accounting for the manufacturer-provided pump efficiency.

**Fig. 2:**
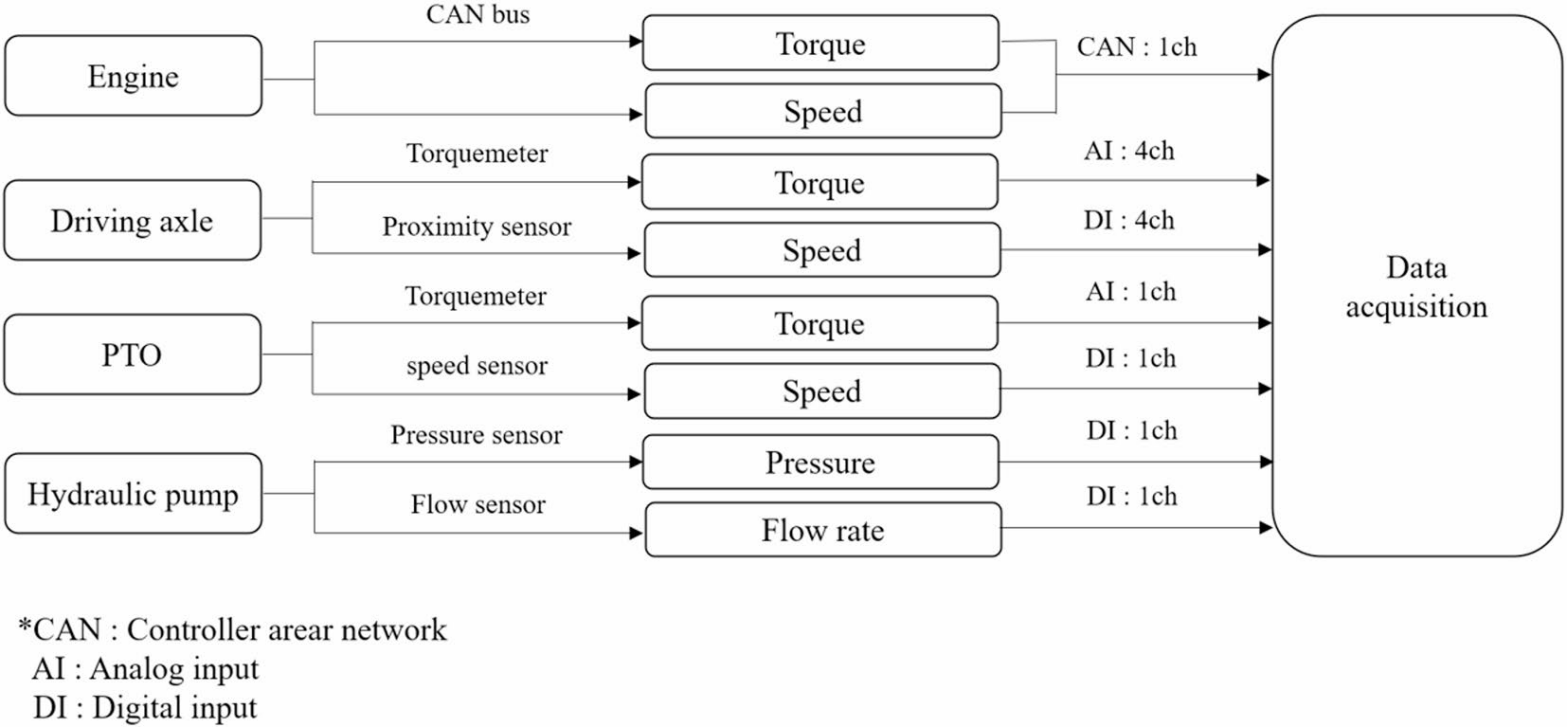
Schematic diagram of the signal flow and data acquisition architecture of the 55-kW agricultural tractor load measurement system.

**Fig. 3:**
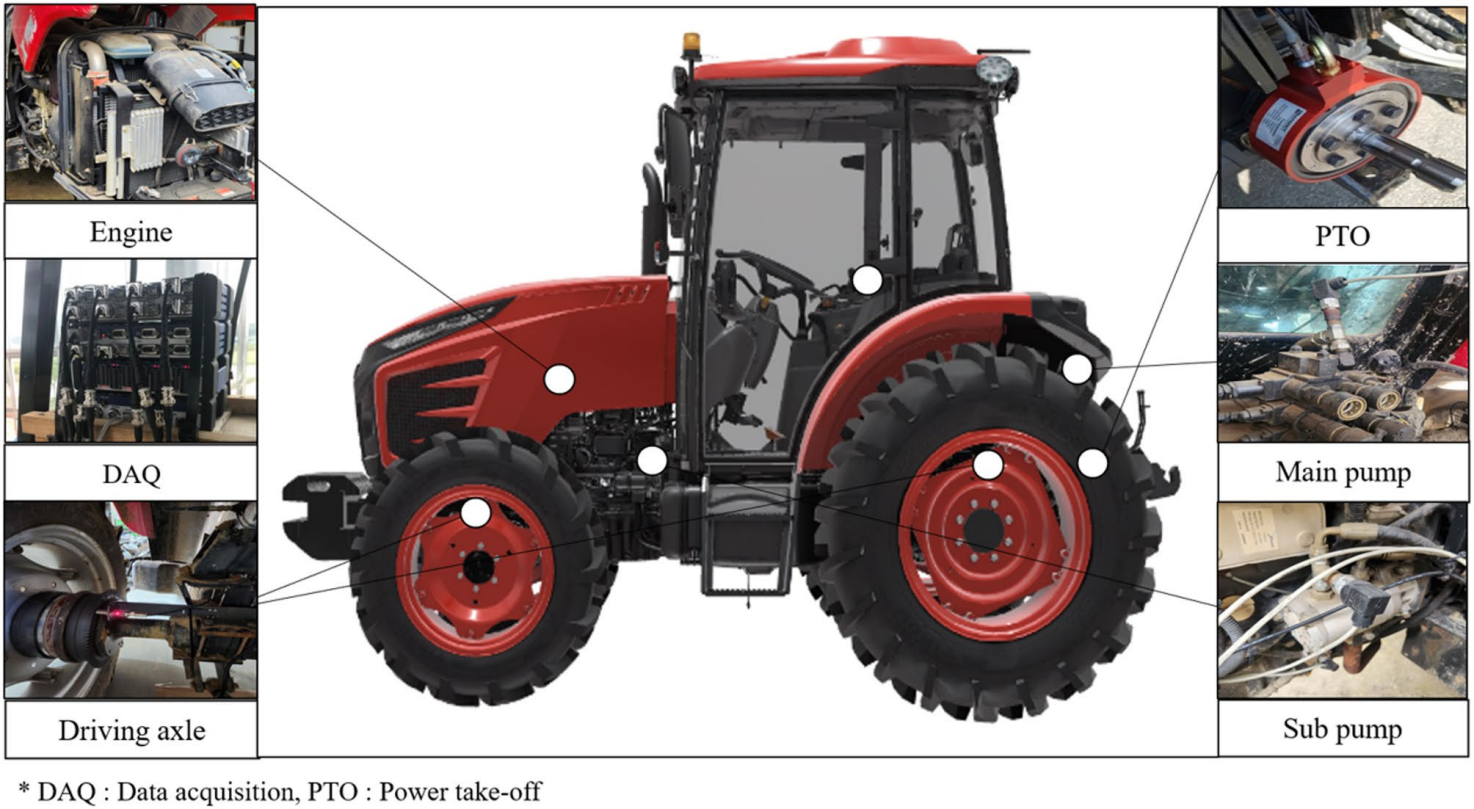
Physical configuration and sensor installation layout of the load measurement system on the 55-kW agricultural tractor.

All signals were simultaneously recorded using a multi-channel data acquisition (DAQ) system to ensure time synchronization among torque, speed, pressure, and flow signals. Zero-offset correction was performed prior to each field experiment to minimize measurement bias. The signal architecture of the measurement system is illustrated in Fig. 2, whereas the physical installation layout of the sensors on the tractor is presented in Fig. 3. Detailed specifications of the sensors and DAQ system are summarized in Table 2.

**Table 2:** Specifications of sensors used in the tractor load measurement system.

Sensor	Model	Manufacturer	Measurement range	Accuracy
DAQ system	QuantumX MX840B	HBM, Germany	8 channels	$\pm$ 0.05%
Axle torquemeter	MW B 30 kNm	Manner, Germany	0–30 kNm	$\pm$ 0.02%
PTO torquemeter	MW B 5kNm	Manner, Germany	0–5 kNm	$\pm$ 0.02%
Proximity sensor	RS-025	DACELL, Korea	Pulse output (12 V DC ± 10%)	-
Pressure sensor	HAD 4400	HYDAC, Germany	0–250 bar	$\pm$ 0.5%
Flow meter	OG2 Oval Gear	Titan Enterprises, UK	0.0–4.0 L/min (30cST oil)	$\pm$ 0.75%

### Field experiment

The field experiment was conducted in a 100 × 40 m paddy field (36° 55′ 49.2″ N, 126° 37′ 58.0″ E) in Korea. Three representative agricultural operations were selected: plow tillage, rotary tillage, and driving operation, which are commonly performed using 55 kW class utility tractors. A six-furrow plow and a rotary tiller were used for tillage operations. The plow required 50–60 kW of tractor power, and the rotary tiller required 48–55 kW. The experiments were repeated three times for each operation under typical working conditions used in Korean farming practice. The gear stages were set to M2 for plow tillage (4.95 km/h), L3 for rotary tillage (2.22 km/h, PTO 540 rpm), and H4 for driving operation (28.26 km/h). The engine was operated at its rated speed of 2200 rpm, and the tillage depth during plow and rotary operations was maintained at 15–20 cm. To ensure data reliability, the experiments were conducted by experienced operators. Among the three repeated trials, the dataset exhibiting the most severe load condition was selected for detailed analysis. The experimental conditions are summarized in Table 3.

**Table 3:** Specifications of agricultural implements used in the field experiment.

Item	Attached implement	
	Plow	Rotavator
Model	WJSP-6S	WJ185A
Manufacturer	Woongjin Machinery, Korea	Woongjin Machinery, Korea
Length $\times$ Width $\times$ Height (mm)	1930 $\times$ 1800 $\times$ 1235	900 $\times$ 2020 $\times$ 1130
Weight (kgf)	370	405
No. of blades	6	42
Working width (mm)	1800	2020
Required power (kW)	50–60	48–55

**Fig. 4:**
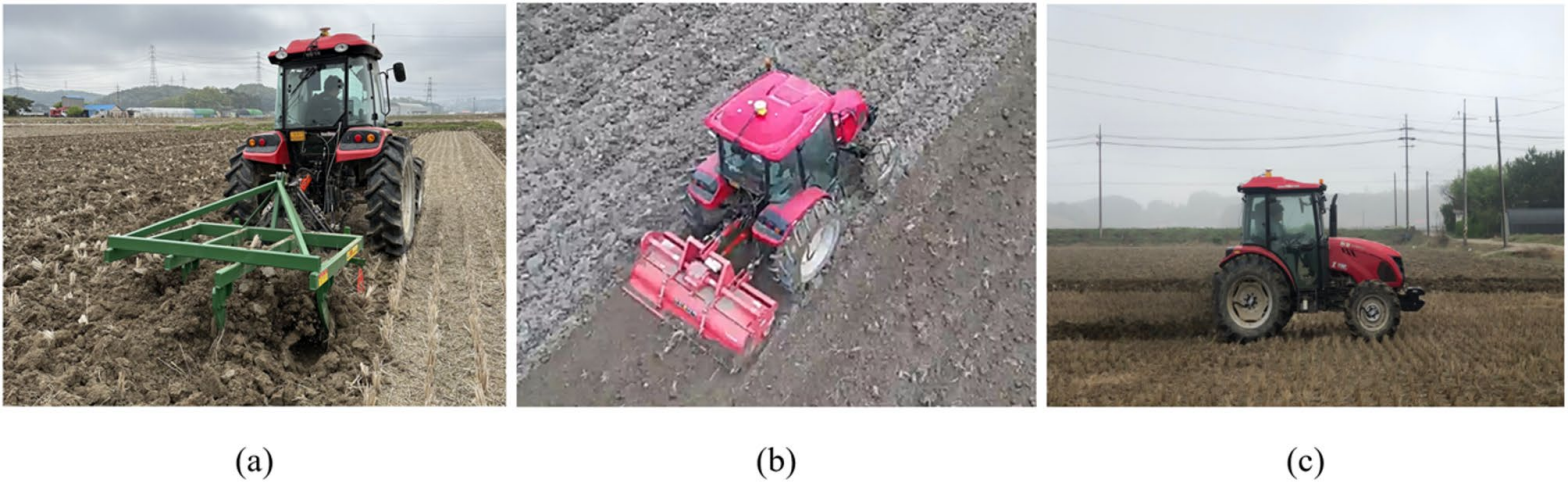
Field experiments for measuring load data during agricultural operations: (**a**) plow tillage, (**b**) rotary tillage, and (**c**) driving operation.

The field experiment was repeated three times for each agricultural operation at typical working speeds used in Korean farming^31^. Gear stages were set as M2 for plow tillage, L3 for rotary tillage, and H4 for driving operation. The engine was operated at its rated speed of 2200 rpm, and the tillage depth during plow and rotary operations was maintained at 15–20 cm. To ensure data reliability, the experiments were conducted by farmers with over five years of field experience. Among the three repeated measurements, the most severe load data was selected for analysis. Detailed field experimental conditions are summarized in Table 4. Figure 4 shows the field experiments for plow tillage, rotary tillage, and driving operation, which were conducted to measure the workload data of the tractor’s main power sources.

**Table 4:** Field experimental conditions used in this study.

Operation	Gear stage (travel speed)	Engine speed (rpm)	PTO speed (rpm)	Depth (cm)
Plow tillage	M2 (4.95 km/h)	2200	–	15–20
Rotary tillage	L3 (2.22 km/h)		540	
Driving operation	H4 (28.26 km/h)		–	–

### Soil condition

**Fig. 5:**
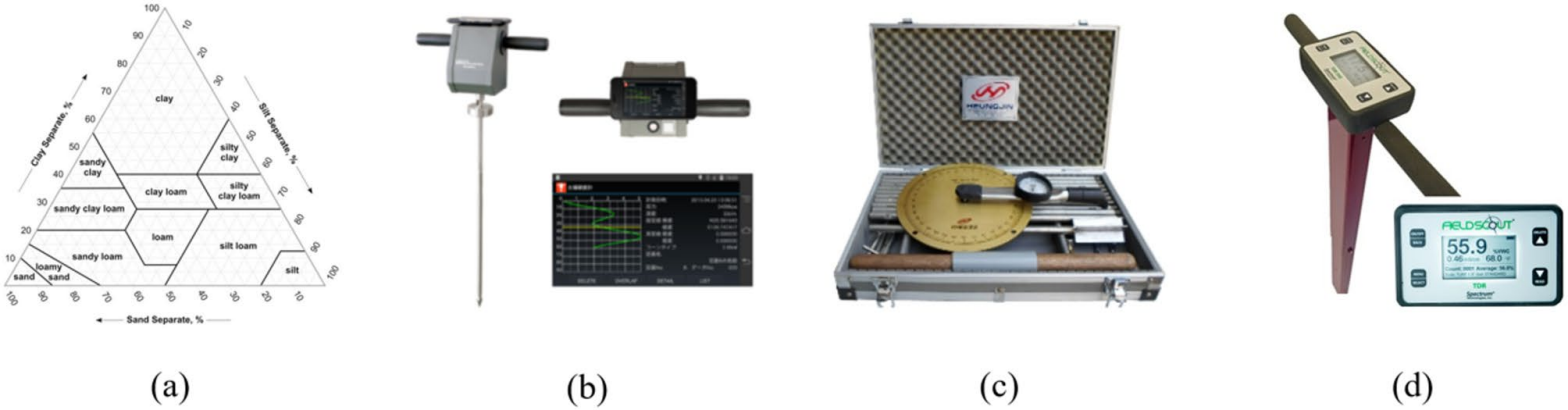
Analysis method of the soil properties: (**a**) soil textural triangle of USDA, (**b**) cone penetrometer, (**c**) potable vane shear apparatus, and (**d**) soil environmental sensor.

Soil properties affecting traction demand and load characteristics were evaluated on the day of the field experiment^32^. Soil texture was classified according to USDA standards, and key mechanical and environmental properties were measured prior to tractor operation. The analysis procedure and instruments used for soil characterization are illustrated in Fig. 5, and the specifications of the soil measurement instruments are summarized in Table 5. Cone penetration resistance was measured to a depth of 200 mm to characterize soil strength distribution with depth. Shear stress was evaluated using a portable vane shear apparatus to estimate soil resistance against torsional deformation. In addition, soil water content, electrical conductivity, and temperature were recorded to assess environmental conditions influencing traction and draft force.

**Table 5:** Specifications of soil measurement instruments used in this study.

Sensor	Model	Manufacturer	Measurement range	Accuracy
Cone penetrometer	DIK-5532	Daiki Rika Kogyo, Japan	0–20,000 kPa	± 3%
Portable vane shear apparatus	HJ-4560	Heungjin Testing Machine, Korea	0–50 kPa	± 2%
Soil environmental sensor	FieldScout TDR350	Spectrum Technologies, USA	Moisture: 0–100% VWC EC: 0–10 dS/m Temperature: − 30 to 60 °C	Moisture: ± 3% VWC EC: ± 0.1 dS/m Temp: ± 0.5 °C

The soil texture was identified as silty loam, consisting of 18.0% sand, 62.2% silt, and 19.8% clay. Cone index values increased with depth, measuring 1,179.4 ± 498.3 kPa at 0–50 mm, 1,424.9 ± 508.6 kPa at 50–100 mm, 1,501.9 ± 446.3 kPa at 100–150 mm, and 2,514.3 ± 657.8 kPa at 150–200 mm. The measured shear stress was 16.2 ± 3.5 kPa. Soil water content, electrical conductivity, and temperature were 17.3 ± 2.1%, 1.0 ± 0.3 dS/m, and 24.0 ± 1.5 °C, respectively. All soil measurements were conducted at 30 sampling points and are reported as mean ± standard deviation. The summarized soil property results are presented in Table 6.

**Table 6:** Soil properties of the field experiment site used in this study.

Item		Value
Soil texture		Silt loam (Sand: 18.0%, Silt: 62.2%, Clay: 19.8%
Cone index (kPa)	0–50 mm	1179.4 $\pm$ 498.3
	50–100 mm	1424.9 $\pm$ 508.6
	100–150 mm	1501.9 $\pm$ 446.3
	150–200 mm	2514.3 $\pm$ 657.8
Shear stress (kPa)		16.2 $\pm$ 3.5
Soil water content (%)		17.3 $\pm$ 2.1
Electric conductivity (dS/m)		1.0 $\pm$ 0.3
Temperature ($^\circ \mathrm{C})$		24.0 $\pm$ 1.5

*Values are presented as mean ± standard deviation.

### Data analysis process

The analysis of tractor power consumption and load spectra was conducted in two steps: (1) calculation of engine, axle, and hydraulic pump power, and (2) generation of LDD and RFC-based load spectra. The engine and individual axle powers were calculated from the measured torque and rotational speed using Eq. (1). The driving axle power was obtained by summing the powers of the four wheels (front-left, front-right, rear-left, and rear-right), as expressed in Eq. (2).

1 $$P = \frac{2\pi{TN}}{{60,000}},$$

2 $$P_{DA} = P_{FL} + P_{FR} + P_{RL} + P_{RR} ,$$

where $P$ is the power (kW), T is the torque (Nm), N is the rotational speed (rpm), $P_{{DA}}$, ${ }P_{FL}$, $P_{FR}$, $P_{LL}$, and $P_{RR}$ are the powers of the total driving axle (kW), front left wheel (kW), front right wheel (kW), rear left wheel (kW), and rear right wheel (kW), respectively.

The hydraulic pump power was calculated from the measured pressure and flow rate as shown in Eq. (3). The total hydraulic power was obtained as the sum of the main and sub pumps using Eq. (4).

3 $$P_{HP} = \eta \frac{pQ}{{600}},$$

4 $$P_{h} = P_{m} + P_{s} ,$$

where $P_{{HP}}$ is the power of the hydruliac pump (kW), ${ }\eta$ is the efficiency of the hydraulic pump (%), $p$ is the pressure of the hydraulic pump (bar), $Q$ is the flow rate of the hydraulic pump (lpm), ${P}_{h}$ is the total power of the hydraulic pump (kW), ${P}_{m}$ is the power of the main pump (kW), and ${P}_{s}$ is the power of the sub pump (kW).

Before calculating the LDD and load spectrum, the measured axle torques and rotational speeds were converted into four-wheel drive (4WD) shaft data using the corresponding gear ratios and transmission efficiencies. This conversion step provides representative shaft-level load data required for electrification analysis. While the mechanical structure of the front and rear axles remains unchanged, the converted torque and speed values are used to replace the functions of the main and range shafts in the electrified configuration. The 4WD axle torque and speed were calculated using Eqs. (5)–(8). The hydraulic pump efficiency, transmission efficiency, and gear ratios used in the calculations were provided by the tractor manufacturer, ensuring consistency in power conversion and load mapping.

5 $$T_{FA} = T_{FL} + T_{FR} ,$$

6 $$T_{RA} = T_{RL} + T_{RR} ,$$

where $T_{FA}$, ${ }T_{FL}$, $T_{FR}$, $T_{RA}$, $T_{RL}$, and $T_{RR}$ are the torques of the front axle (Nm), front left wheel (Nm), and front right wheel (Nm), rear axle (Nm), rear left wheel (Nm), and rear right wheel (Nm), respectively.

7 $$T_{4WD} = T_{FA} \ i_{FA\_4WD} \eta_{FA\_4WD} + T_{RA} \ i_{RA\_4WD} \eta_{RA\_4WD} ,$$

8 $$N_{4WD} = \frac{{N_{FA}}}{\ i_{FA\_4WD}} = \frac{{N_{RA}}}{\ i_{RA\_4WD}},$$

where $T_{4WD}$ is the torque of the 4WD axle (Nm), $i_{FA\_4WD}$ is the gear ratio for the front axle to the 4WD axle, $\eta_{{FA\_4WD}}$ is the efficiency from the front axle to the 4WD axle (%), $i_{RA\_4WD}$ is the gear ratio for the rear axle to 4WD axle, $\eta_{{RA\_4WD}}$ is the efficiency from the rear axle to the 4WD axle (%), $N_{4WD}$ is the rotational speed of the 4WD axle (rpm), and $N_{FA}, N_{RA}$ are the rotational speed of the front and rear axle (rpm).

**Fig. 6:**
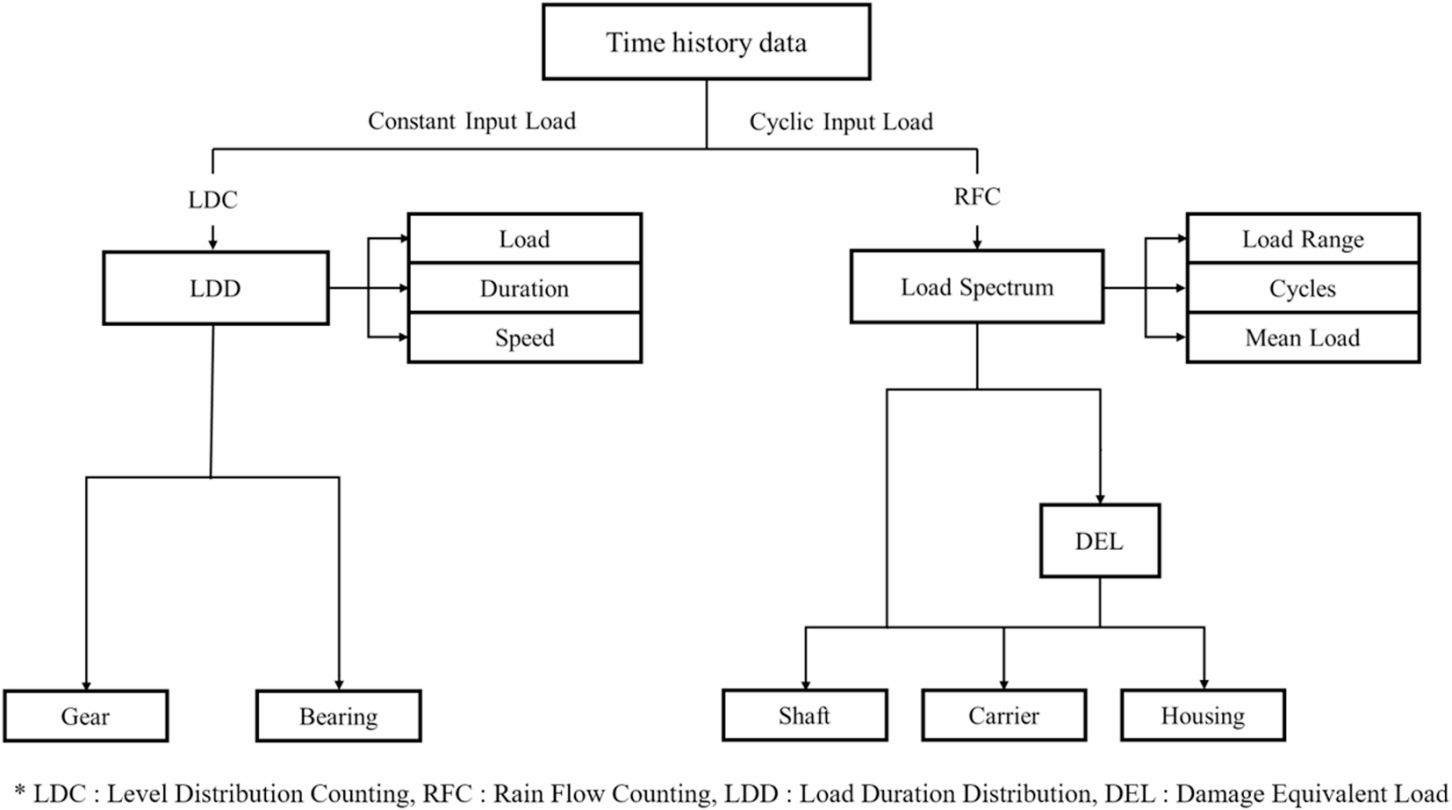
Data analysis process for level distribution counting and rainflow counting.

The processed torque-time data were then used to generate two types of load information: LDD and RFC. The LDD quantifies the distribution of torque levels with respect to their exposure duration and rotational speed, providing information on how long specific load ranges act on drivetrain components. This data is particularly useful for gears and bearings, which are primarily affected by the magnitude and duration of applied loads during operation. In contrast, the RFC method converts the torque-time history into a series of closed load cycles, capturing both the range and mean of each cycle. This cyclic load information is essential for fatigue life evaluation of rotating components such as shafts, carriers, and housings, which are more sensitive to cyclic stress amplitudes than to static load magnitude. Figure 6 illustrates the overall process of converting the measured time-history torque data into LDD and RFC-based load spectra for powertrain durability analysis. The torque range between the minimum and maximum measured values was divided into discrete load bins. For each bin, the average torque, total exposure time, and average rotational speed were calculated using Eqs. (9)–(11). The structured LDD and RFC outputs can serve as design-oriented reference data for component rating verification, fatigue assessment, and the development of representative evaluation cycles in electrified tractor systems^33^.

9 $$T_{i} = \frac{{\mathop \sum \nolimits_{j = 1}^{n} T_{i,j} }}{n},$$

10 $$t_{i} = {\Delta }t \cdot n,$$

11 $$N_{i} = \frac{{\mathop \sum \nolimits_{j = 1}^{n} N_{i,j} }}{n},$$

where i is the bin index, $T_{i}$ is the average torque in the *i*th bin (Nm), $T_{i,j}$ is the *j*th torque sample in the *i*th bin (Nm), n is the number of data samples in the bin, t is the time interval of the measurement data (s), $N_{i}$ is the average rotational speed of the *i*th bin (rpm), and $N_{i,j}$ is the *j*th rotational speed samples in the *i*th bin (rpm).

## Results

### Measured workload data

**Fig. 7:**
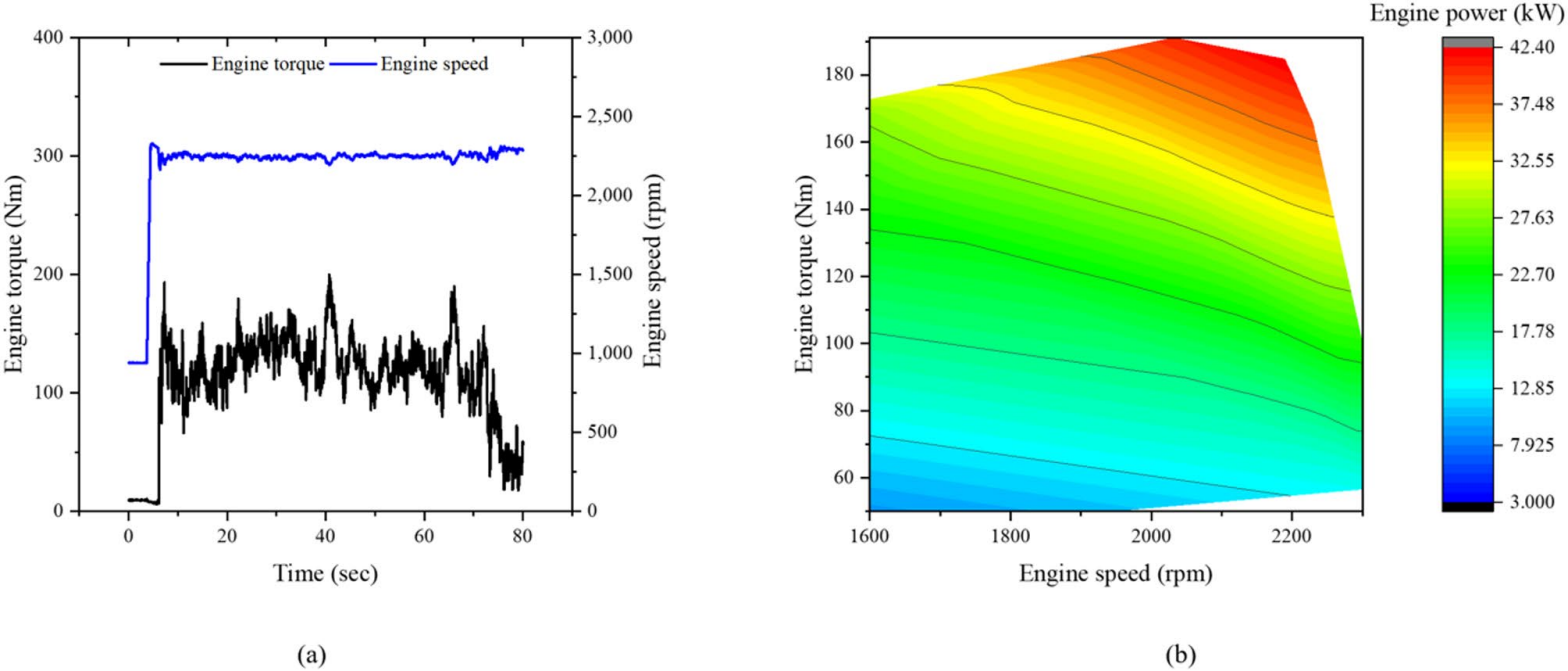
Engine characteristics of the tractor during plow tillage: (**a**) time history of engine torque and rotational speed, and (**b**) torque–speed operating region with corresponding engine power contour.

Figure 7a shows the measured time histories of engine torque and rotational speed during plow tillage at the M2 gear stage, while Fig. 7b illustrates the torque–speed distribution and corresponding engine power contour, which defines the engine operating region under this workload condition. During the working section, the engine torque, speed, and power ranged from 110.1 to 199.8 Nm, 2184.6 to 2328.0 rpm, and 25.8 to 45.9 kW, respectively. These correspond to 45.9–83.2% of the rated engine torque (300 Nm) and 46.7–83.1% of the rated engine power (55.3 kW), respectively, indicating that the engine primarily operated under medium to high load conditions during plow tillage.

**Fig. 8:**
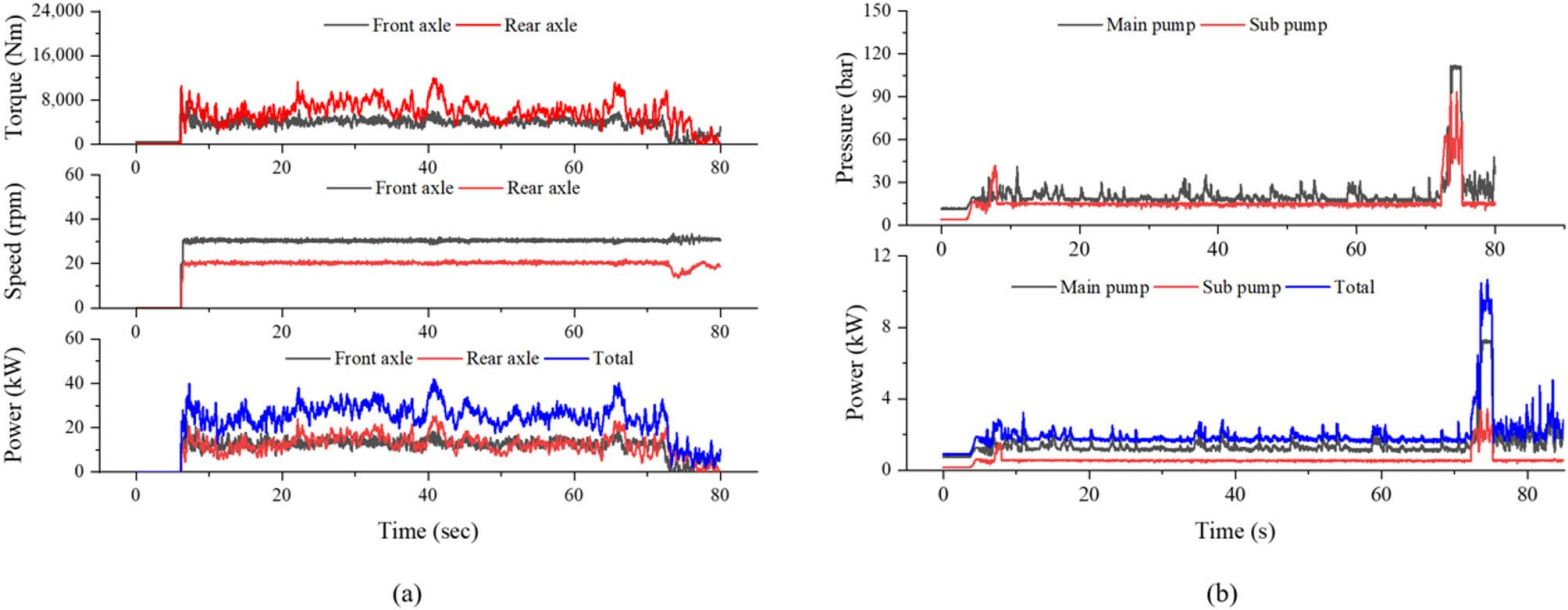
Driving axle and hydraulic pump data of the tractor during plow tillage: (**a**) axle torque, speed, and power, and (**b**) hydraulic pump pressure and power.

Figure 8a presents the driving axle data at the same operating condition. The front axle torque, speed, and power ranged from 3552.5 to 10,012.5 Nm, 28.1 to 33.5 rpm, and 11.2 to 24.2 kW, respectively, while the rear axle ranged from 5405.0 to 12,008.9 Nm, 18.5 to 21.9 rpm, and 11.4 to 25.3 kW. The total driving axle power ranged from 22.5 to 41.9 kW. Overall, the rear axle transmitted higher torque at a lower rotational speed, whereas the front axle rotated faster with slightly lower torque, reflecting the typical torque distribution pattern of a 4WD tractor during plow tillage. Figure 8b shows the hydraulic pump data for the same operation. The main pump pressure and power ranged from 22.1 to 111.9 bar and 1.4 to 7.3 kW, respectively, while the sub pump ranged from 16.1 to 93.2 bar and 0.6 to 3.4 kW. The total hydraulic pump power varied from 2.0 to 10.7 kW, which was considerably lower than the axle power, indicating that traction requirement dominated the overall power consumption during plow tillage.

**Fig. 9:**
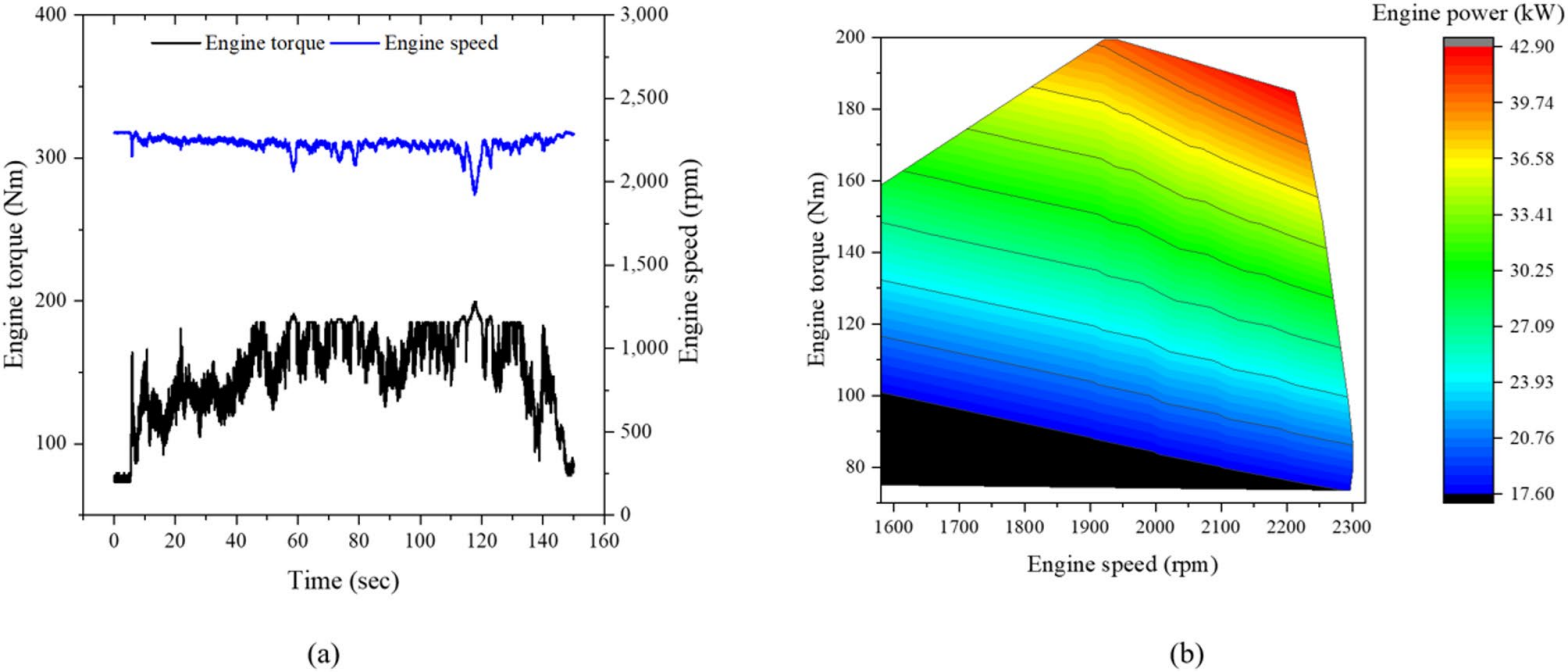
Engine characteristics of the tractor during rotary tillage: (**a**) time history of engine torque and rotational speed, and (**b**) torque–speed operating region with corresponding engine power contour.

Figure 9 shows the engine torque, speed, and power of the tractor during rotary tillage at the L3 gear stage. In the working section, the engine torque, speed, and power ranged from 150.7 to 199.5 Nm, 2225.1 to 2301.5 rpm, and 35.0 to 42.8 kW, respectively. These correspond to 62.8–83.2% of the rated torque and 46.7–83.1% of the rated power, indicating that the engine operated at a relatively higher load fraction compared with plow tillage.

**Fig. 10:**
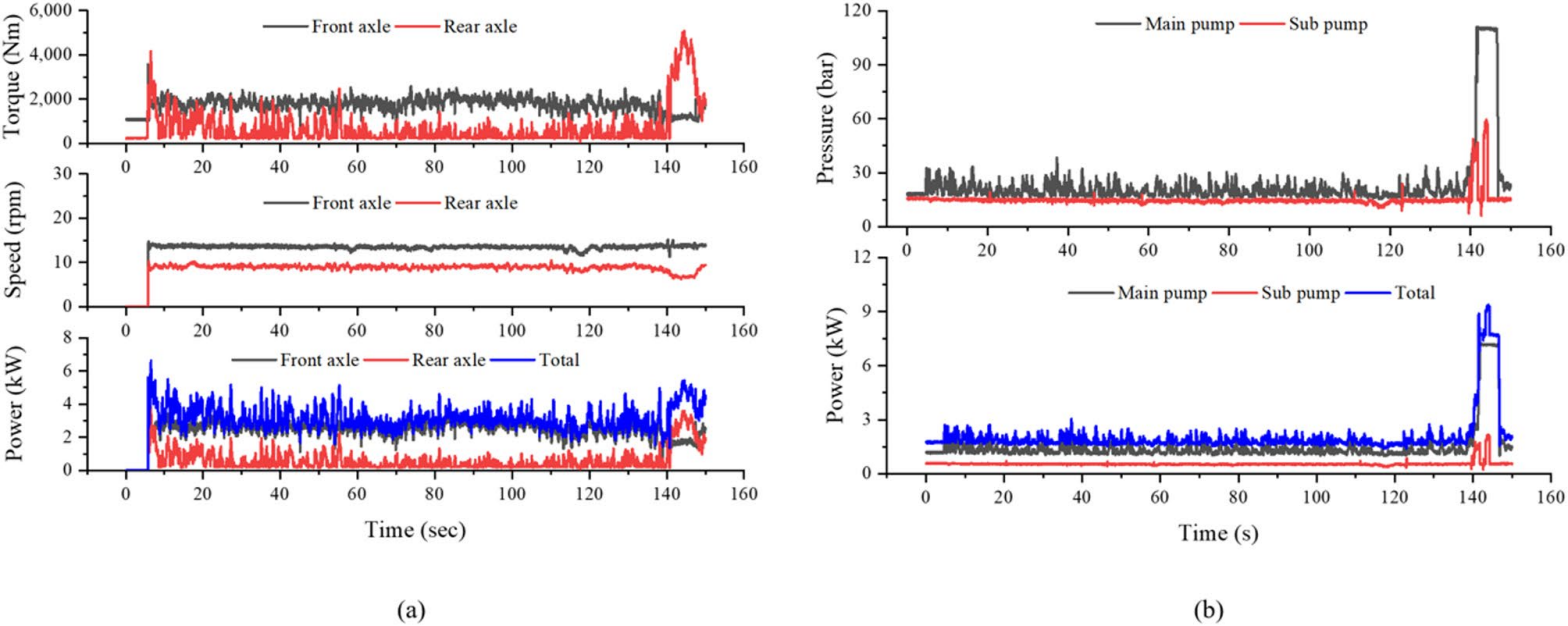
Driving axle and hydraulic pump data of the tractor during rotary tillage: (**a**) axle torque, speed, and power, and (**b**) hydraulic pump pressure and power.

Figure 10a presents the driving axle data under the same condition. The front axle torque, speed, and power ranged from 1746.7 to 3558.0 Nm, 13.0 to 15.2 rpm, and 2.4 to 4.0 kW, respectively, while the rear axle ranged from 547.3 to 4162.0 Nm, 8.6 to 10.2 rpm, and 0.5 to 3.8 kW. The total driving axle power ranged from 3.0 to 6.6 kW. Compared with plow tillage, the overall axle power was significantly lower, reflecting the lighter traction requirement of rotary tillage where the implement is primarily powered through the PTO. Figure 10b shows the hydraulic pump data during rotary tillage. The main pump pressure and power ranged from 23.6 to 111.2 bar and 1.5 to 7.2 kW, respectively, while the sub pump ranged from 15.2 to 59.5 bar and 0.6 to 2.2 kW. The total hydraulic pump power ranged from 2.1 to 9.4 kW, slightly higher than during plow tillage, due to continuous implement lifting and hydraulic control during rotary operation.

**Fig. 11:**
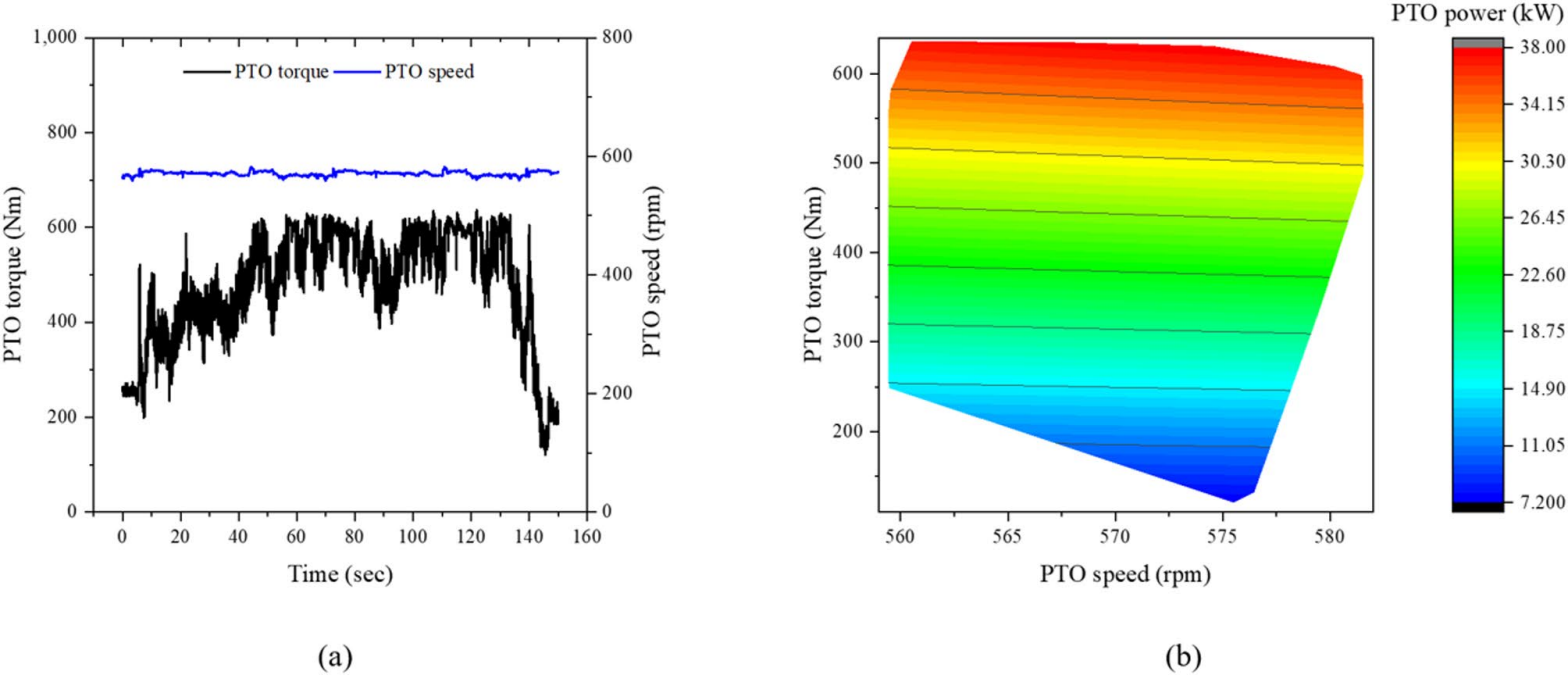
PTO characteristics of the tractor during rotary tillage: (**a**) time history of PTO torque and rotational speed, and (**b**) torque–speed operating region with corresponding PTO power contour.

Figure 11 presents the PTO data of the tractor during rotary tillage. The PTO torque, speed, and power ranged from 476.2 to 636.0 Nm, 570.9 to 581.6 rpm, and 28.5 to 38.0 kW, respectively. The PTO power corresponded to 51.5–68.7% of the rated engine power during this operation, indicating that rotary tillage is predominantly PTO-driven with minimal traction load on the driving axles.

**Fig. 12:**
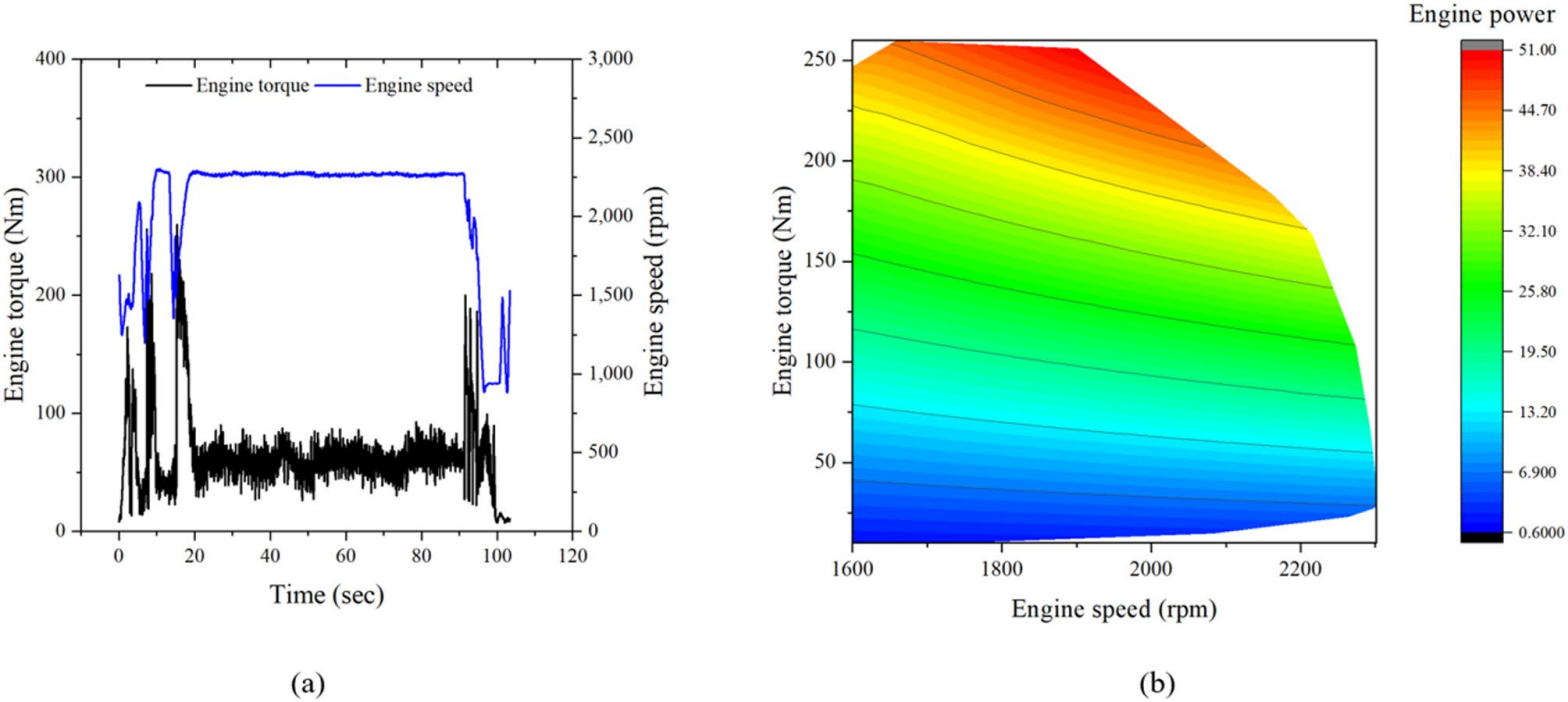
Engine characteristics of the tractor during driving operation: (**a**) time history of engine torque and rotational speed, and (**b**) torque–speed operating region with corresponding engine power contour.

Figure 12 shows the engine torque, speed, and power of the tractor during driving operation at the H4 gear stage. During the working section, the engine torque, speed, and power ranged from 63.7 to 259.7 Nm, 2082.2 to 2301.5 rpm, and 14.0 to 51.0 kW, respectively. The engine torque and power corresponded to 26.6–108.2% and 25.3–92.1% of the rated values, indicating that the engine experienced frequent load fluctuations, including short periods near full load, during driving operation.

**Fig. 13:**
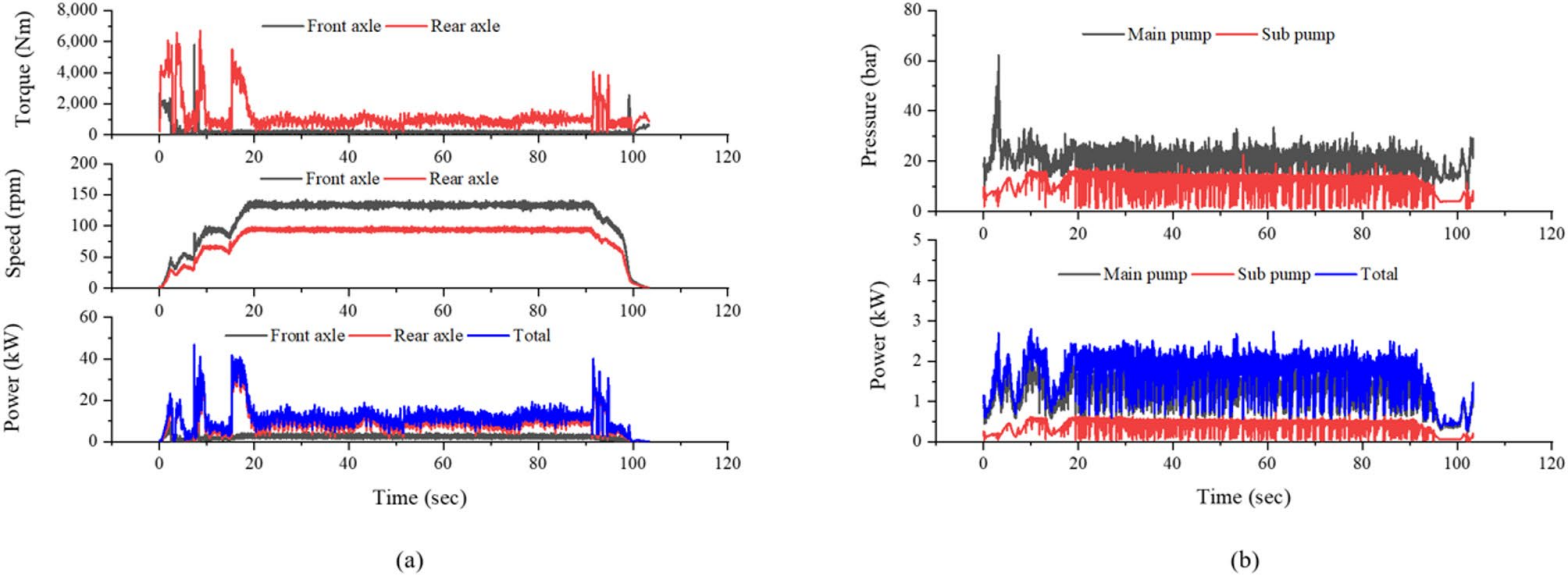
Driving axle and hydraulic pump data of the tractor during driving operation: (**a**) axle torque, speed, and power, and (**b**) hydraulic pump pressure and power.

Figure 13a presents the driving axle data under the same condition. The front axle torque, speed, and power ranged from 247.6 to 5792.0 Nm, 114.4 to 142.8 rpm, and 2.4 to 42.8 kW, respectively. The rear axle ranged from 1205.5 to 6690.0 Nm, 80.5 to 99.8 rpm, and 9.2 to 39.5 kW. The total driving axle power ranged from 11.6 to 46.9 kW. The rear axle carried the majority of the traction load, while the front axle contributed mainly at higher speeds, reflecting the typical load distribution of 4WD tractors during driving operation on unpaved terrain. Figure 13b shows the hydraulic pump data during driving operation. The main pump pressure and power ranged from 20.9 to 62.1 bar and 1.3 to 2.6 kW, respectively, while the sub pump ranged from 11.5 to 22.5 bar and 0.4 to 0.8 kW. The total hydraulic pump power ranged from 1.7 to 3.4 kW, which was minimal compared with axle power, confirming that traction dominated the power requirement during driving operation.

### Results of power requirement

**Fig. 14:**
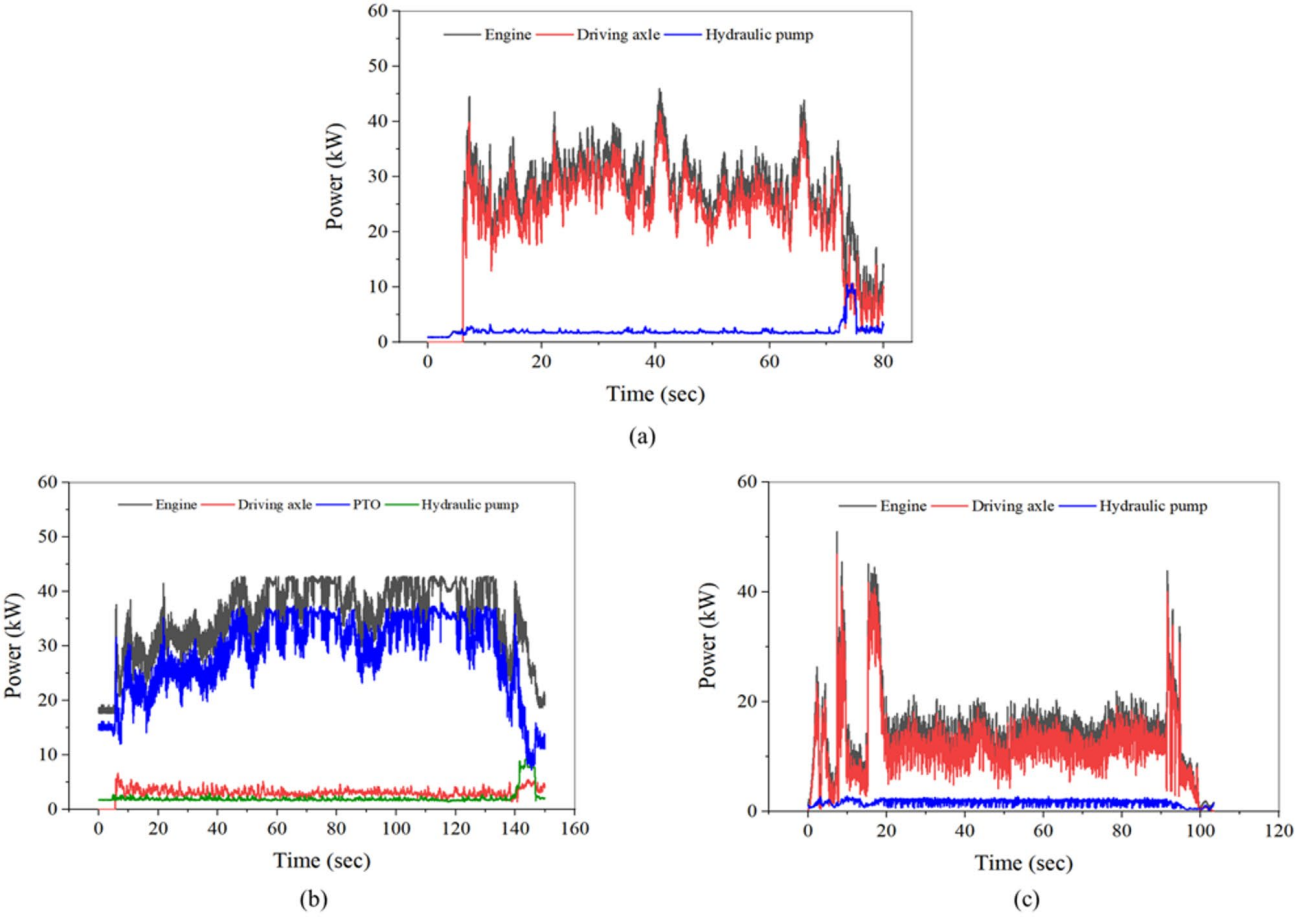
Calculated power requirements for the tractor during representative agricultural operations: (**a**) plow tillage, (**b**) rotary tillage, and (**c**) driving operation.

Figure 14 present the calculated power requirements of the engine, driving axle, PTO, and hydraulic pump during three representative agricultural operations: plow tillage (M2 gear stage), rotary tillage (L3 gear stage), and driving operation (H4 gear stage). During plow tillage, the engine, driving axle, and hydraulic pump required maximum powers of 45.9 kW, 41.9 kW, and 10.7 kW, respectively, with corresponding average powers of 25.8 kW, 22.5 kW, and 2.0 kW. The total tractor power peaked at 52.6 kW, corresponding to 95.1% of the rated engine power. Power requirement was dominated by the driving axle, whereas the hydraulic pump contributed minimally, confirming that plow tillage is primarily traction-driven. During rotary tillage, the engine, driving axle, PTO, and hydraulic pump required maximum powers of 42.8 kW, 6.6 kW, 34.4 kW, and 2.7 kW, respectively. Their average powers were 30.5 kW, 3.0 kW, 28.5 kW, and 2.1 kW. The total power peaked at 45.3 kW (81.9% of rated power), and the PTO accounted for the majority of the engine load, while the axle load remained low. During driving operation, the engine, driving axle, and hydraulic pump required maximum powers of 51.0 kW, 46.9 kW, and 2.8 kW, respectively. The corresponding average powers were 14.5 kW, 12.0 kW, and 1.8 kW. The total power reached 49.6 kW (90.2% of rated power) during transient acceleration and traction peaks, while hydraulic requirement remained negligible. This indicates that driving operation is characterized by intermittent traction load with low hydraulic requirement.

The detailed statistics of the maximum and average power requirements are summarized in Table 7. Both the maximum and average percentages represent values relative to the rated engine power of 55.3 kW. Plow tillage exhibited the highest peak total power of 52.6 kW, with the driving axle contributing the dominant share of the total load. Rotary tillage showed a PTO-dominated pattern, where axle power remained below 6.6 kW. Driving operation demonstrated lower average power but relatively high peak values, reflecting transient traction events. Across all operations, hydraulic pump power remained consistently low, with an average of less than 3 kW, confirming that hydraulic demand was minimal compared to traction and PTO loads. Overall, clear differences in subsystem power distribution were observed among operation types: plow tillage was traction-dominated, rotary tillage was PTO-driven, and driving operation exhibited intermittent traction load with minimal hydraulic contribution.

**Table 7:** Calculated maximum and average power requirements for the tractor during representative agricultural operations.

Operation	Item	Power requirement			
		Max. (kW)	Max. (%)	Avg. (kW)	Avg. (%)
Plow tillage	Engine	45.9	83.0	25.8	46.7
	Axle	41.9	75.8	22.5	40.7
	Hydraulic pump	10.7	19.3	2.0	3.6
	Total	52.6	95.1	24.6	44.5
Rotary tillage	Engine	42.5	76.9	30.5	55.2
	Axle	6.6	14.8	3.0	5.4
	PTO	34.4	62.2	28.5	51.5
	Hydraulic pump	2.7	4.9	2.1	3.8
	Total	45.3	81.9	32.2	58.2
Driving operation	Engine	51.0	92.7	14.5	26.4
	Axle	46.9	85.3	12.0	21.8
	Hydraulic pump	2.8	5.1	1.8	3.3
	Total	49.6	90.2	13.8	25.1

*Max. and Avg. (%) indicate values relative to the rated engine power of 55.3 kW.

**Fig. 15:**
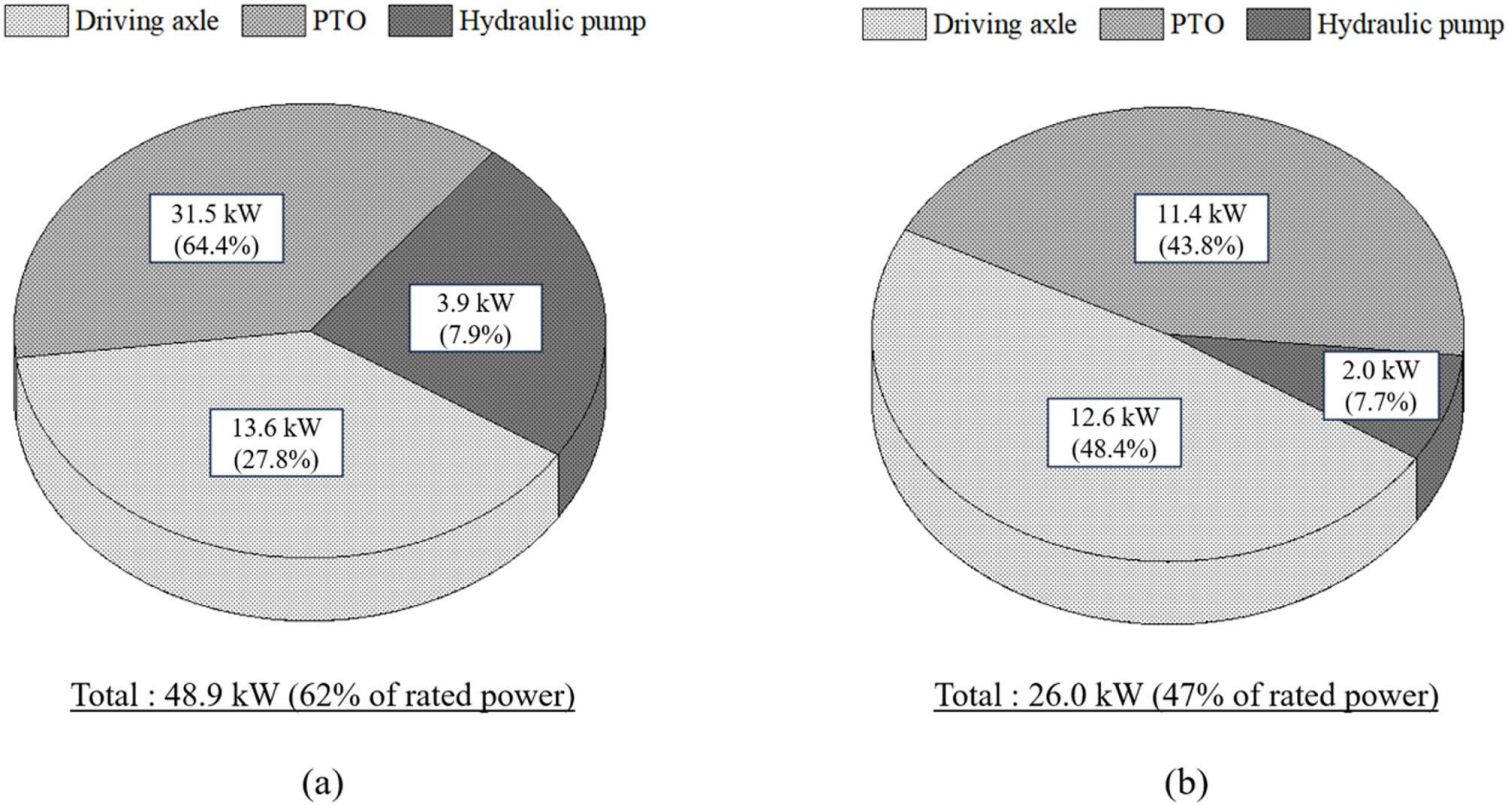
Comparison of power requirements for tractor major parts between previous and this study: (**a**) results of 78-kW tractor, and (**b**) results of 55-kW tractor.

Figure 15 compares the power requirements of major tractor components between the present 55-kW tractor and a previously studied 78-kW tractor^34^. The total power requirement of the 78-kW tractor was 48.9 kW, corresponding to 62% of its rated engine power, while the 55-kW tractor required 26.0 kW in total (47% of rated power). The 78-kW tractor exhibited a broader output range and higher total requirement because it is designed for compact, high-load operations including moldboard plow, disk plow, rotary tillage, and baler operation.

For the 78-kW tractor, the power requirements of the driving axle, PTO, and hydraulic pump were 13.6 kW (27.8%), 31.5 kW (64.4%), and 3.9 kW (7.9%), respectively. In the 55-kW tractor, the corresponding values were 12.6 kW (48.4%), 11.4 kW (43.8%), and 2.0 kW (7.7%). The hydraulic pump ratio was similar in both tractors, at approximately 7%, but the PTO share was about 20 percentage points higher in the 78-kW tractor, reflecting the greater proportion of PTO-driven operations such as baler work. Overall, the comparison confirms that the 55-kW tractor exhibits a lower total power requirement and a traction-dominant distribution, whereas the 78-kW tractor shows a PTO-dominant load profile. This highlights the importance of considering implement mix and duty cycles when evaluating tractors of different rated outputs.

### Results of data processing

**Fig. 16:**
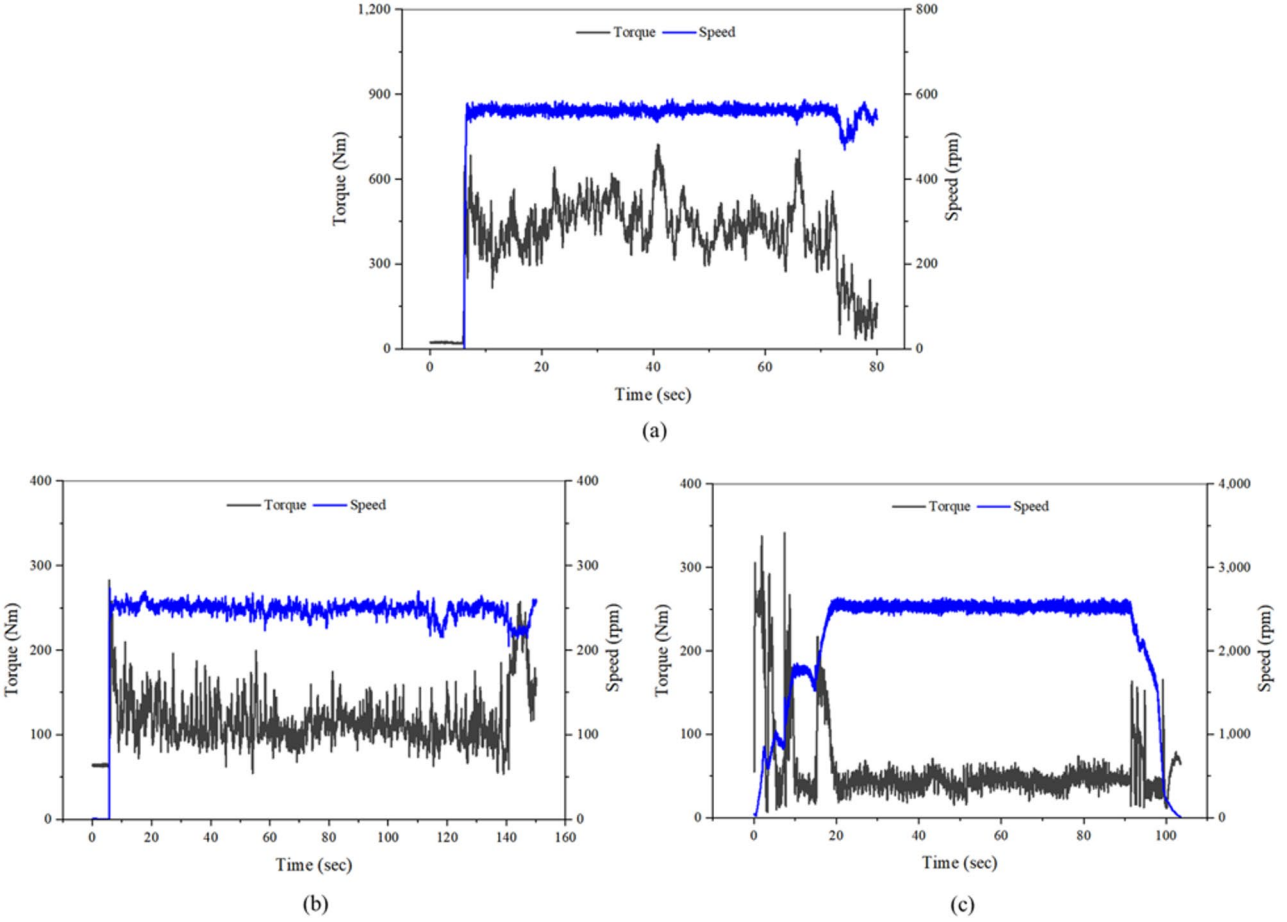
Calculated 4WD axle torque and rotational speed during representative agricultural operations: (**a**) plow tillage, (**b**) rotary tillage, and (**c**) driving operation.

Figure 16 shows the 4WD axle torque and rotational speed converted from the measured wheel data during three representative agricultural operations. During plow tillage, the torque ranged from 19.8 to 723.8 Nm and the rotational speed from 151.0 to 565.0 rpm, indicating sustained traction demand under relatively moderate rotational speeds. Rotary tillage showed a lower torque range (54.0–283.0 Nm) and a rotational speed of 121.0–249.4 rpm, reflecting relatively stable PTO-driven operation with limited traction variation. In contrast, driving operation exhibited highly fluctuating torque (6.96–341.7 Nm) at 388.4–2304.6 rpm, reflecting intermittent traction peaks and frequent low-load or coasting phases associated with speed transitions. These operational differences directly influence the load case distribution observed in the LDD results and contribute to the variation in cyclic load characteristics considered in the subsequent RFC-based analysis.

Table 8 summarizes the LDD results for the 4WD axle, classified into eight load cases according to torque, speed, duration, and frequency. Plow tillage showed the highest load concentration in the 5th case (418.8 Nm at 563.5 rpm), which accounted for 33.9% of the total duration. Rotary tillage was dominated by the 3rd load case (99.3 Nm at 249.3 rpm), representing 45.1% of the duration, while driving operation was concentrated in the 1st load case (38.6 Nm at 2304.6 rpm), occupying 60.6% of the total duration. These results indicate that traction-based operations such as plow tillage are concentrated at mid-to-high torque with moderate rotational speeds, while PTO-based operations such as rotary tillage maintain relatively low torque and operate under steady conditions. In contrast, driving operation is dominated by low-torque conditions with long periods of coasting or light load.

**Table 8:** Results of the LDD for the 4WD shaft during representative agricultural operations.

Load case	Plow tillage				Rotary tillage				Driving operation			
	Torque (Nm)	Speed (rpm)	Duration (s)	Frequency (%)	Torque (Nm)	Speed (rpm)	Duration (s)	Frequency (%)	Torque (Nm)	Speed (rpm)	Duration (s)	Frequency (%)
1	38.0	151.0	8.3	10.4	69.8	121.0	10.7	7.2	38.6	2304.6	62.7	60.6
2	150.4	531.2	3.1	3.9	99.3	249.3	67.6	45.1	57.7	2177.0	29.4	28.4
3	239.6	521.9	2.2	2.7	122.2	248.7	45.7	30.5	111.3	1850.4	3.4	3.2
4	341.0	565.0	11.6	14.5	151.7	248.9	15.8	10.5	153.3	1897.0	3.4	3.3
5	418.8	563.5	27.1	33.9	180.1	240.0	4.8	3.2	192.8	1145.6	1.7	1.6
6	496.8	560.6	20.5	25.7	210.9	224.6	2.9	2.0	242.8	512.2	1.7	1.6
7	581.7	545.9	5.5	6.9	237.7	221.4	2.2	1.5	270.7	388.4	1.0	0.9
8	665.0	548.8	1.6	2.0	257.9	226.1	0.1	0.1	316.2	462.7	0.2	0.2
Sum	–	–	79.9	100.0	–	–	153.9	100.0	–	–	103.4	100.0

**Fig. 17:**
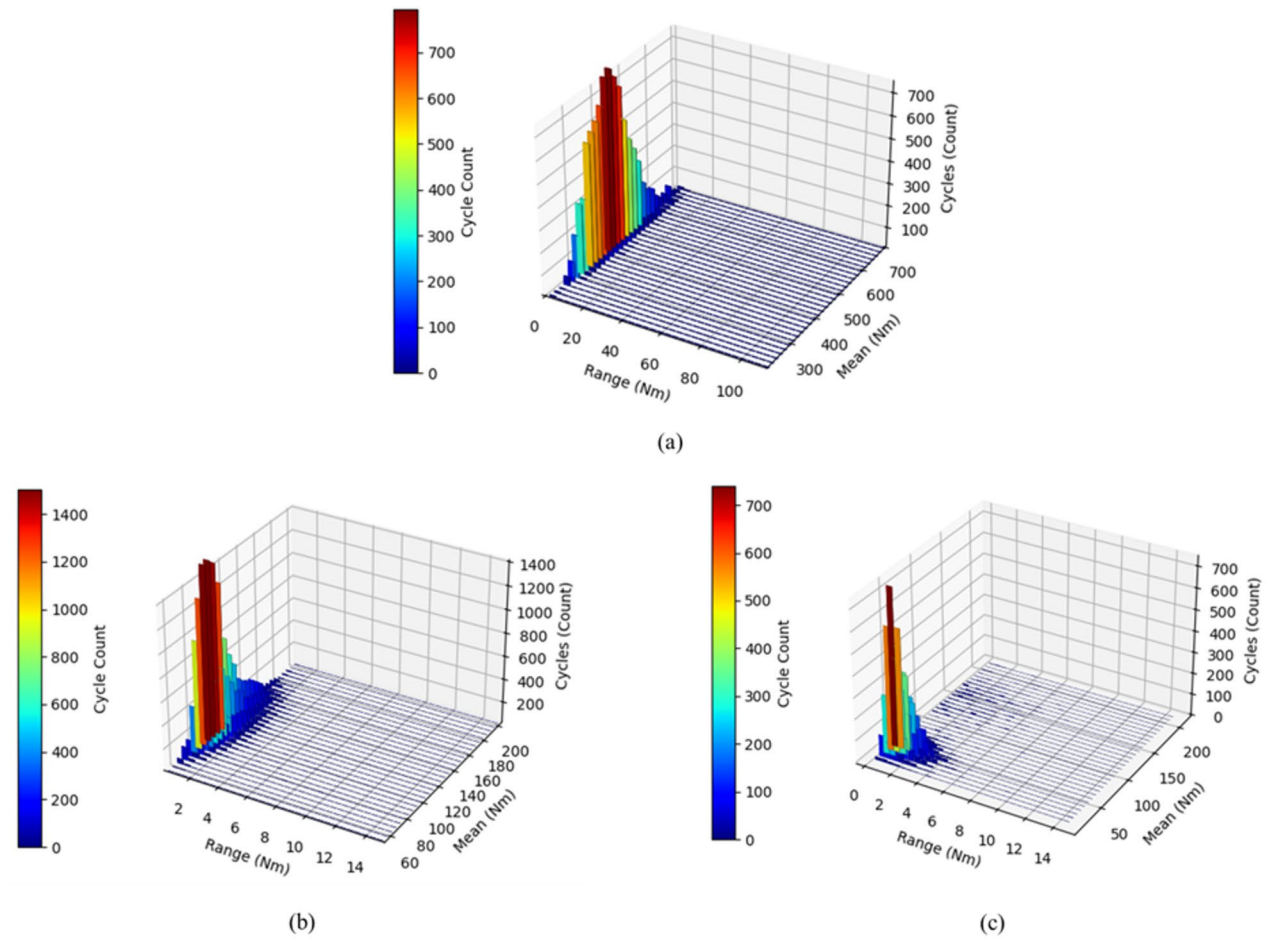
Results of the load spectra for the 4WD shaft during representative agricultural operations: (**a**) plow tillage, (**b**) rotary tillage, and (**c**) driving operation.

Figure 17 illustrates the load spectra of the 4WD axle obtained using the RFC method. Plow tillage exhibited the highest number of cycles in the 500–600 Nm mean range with load amplitudes of 0–20 Nm. Rotary tillage showed the highest cycle count at 100–140 Nm and 0–2 Nm load amplitude, and driving operation presented its peak cycles in the 30–70 Nm mean range with minimal amplitudes (0–2 Nm). These findings confirm that cyclic loads are most severe in traction-dominant operations, whereas rotary and driving operations primarily experience low-amplitude cycles, which has direct implications for the fatigue life assessment and design of axles and rotating components.

## Conclusions

This study established a field-data-based methodology for quantifying power requirements and deriving representative design loads for the electrification of a 55-kW agricultural tractor. The analysis integrated measured torque and speed data from the engine, driving axles, PTO, and hydraulic pump under representative agricultural operations, and generated LDD- and RFC-based load spectra for durability-oriented assessment.

The power requirement analysis revealed that the 55-kW tractor exhibits a lower overall power demand compared with previously studied higher-powered tractors and demonstrates a relatively balanced distribution between traction and PTO loads, while hydraulic power demand remains comparatively small and stable. These findings indicate that electrified powertrain design should be based on subsystem-level power distribution rather than solely on rated engine power, providing quantitative reference values for motor capacity selection and system-level powertrain configuration.

The LDD and RFC analyses further identified duty-cycle characteristics and fatigue-critical load conditions. The LDD results showed that a limited number of load ranges dominate operational exposure, while the RFC-derived load spectra indicated that although low-amplitude cycles are prevalent, high-load cycles are concentrated during tillage operations. These load characteristics provide engineering input for gear and shaft load rating, bearing life estimation, and durability evaluation of structural components such as carriers and housings. In addition, the measured hydraulic power requirements under different operating conditions provide reference data for evaluating hydraulic pump capacity and energy management strategies in electrified tractor platforms.

Overall, the proposed methodology offers a practical design-oriented reference for motor sizing considerations, component strength evaluation, and durability validation of utility electric tractor powertrains. Although this study focused on plow tillage, rotary tillage, and driving operations—representative tasks in Korean agriculture—future work will incorporate a broader range of field operations to construct a more comprehensive load spectrum, support the development of standardized test codes, and further validate the durability and efficiency of electrified tractor systems under diverse real-world conditions.

## Data Availability

The datasets used and analyzed during the current study available from the corresponding author, [Y.J.K.], on reasonable request.

## References

[1] Gong, S. Y. et al. Prediction of tractor drawbar pull under different tillage tools using machine learning and low-cost sensors. *Sci. Rep.* **15** (2025). 10.1038/s41598-025-24974-w PMC12635318 41266736

[2] Do, Y. W. et al. Determining exhaust emissions (CO, NOx, PM) for a combine harvester based on measured engine load and emission factors using PEMS during actual field operation. *Comput. Electron. Agric.* **231**, 110026 (2025).

[3] Lee, S. E. et al. Load factor evaluation and emissions calculation for the cultivator under real working conditions. *J. Agric. Food Res.* **19** (2025).

[4] Lajunen, A., Sainio, P., Laurila, L., Pippuri-Mäkeläinen, J. & Tammi, K. Overview of powertrain electrification and future scenarios for non-road mobile machinery. *Energies* **11** (2018).

[5] Zhang, S.-li et al. A joint control method considering travel speed and slip for reducing energy consumption of rear wheel independent drive electric tractor in ploughing. *Energy* **263**, 126008 (2023).

[6] Ahn, D. V. et al. Development and performance evaluation of a PTO-based power assist system to improve traction force for electric tractors. *Sci. Rep*. **16** (2026). 10.1038/s41598-025-31465-5 PMC12804872 41372370

[7] Baek, S. M., Jeon, H. H., Kim, W. S., Kim, Y. S. & Kim, Y. J. Design and analysis of a power transmission system for 55 kW electric tractor using agricultural workload data. *Sci. Rep.* **15**, 1–17 (2025). 10.1038/s41598-025-11444-6 PMC12313879 40745442

[8] Hensh, S., Tewari, V. K. & Upadhyay, G. An instrumentation system to measure the loads acting on the tractor PTO bearing during rotary tillage. *J. Terramechanics* **96**, 1–10 (2021).

[9] Kumar, A. A., Tewari, V. K. & Nare, B. Embedded digital draft force and wheel slip indicator for tillage research. *Comput. Electron. Agric*. **127**, 38–49 (2016).

[10] Jeon, H. H. et al. Efficiency analysis of powertrain for internal combustion engine and hydrogen fuel cell tractor according to agricultural operations. *Sensors* **24** (2024). 10.3390/s24175494 PMC11398181 39275405

[11] Baek, S. M., Kim, W. S., Kim, Y. S., Baek, S. Y. & Kim, Y. J. Development of a simulation model for HMT of a 50 kW class agricultural tractor. *Appl. Sci*. **10**, 4064 (2020).

[12] Lajunen, A., Kivekäs, K., Freyermuth, V., Vijayagopal, R. & Kim, N. Simulation-based assessment of energy consumption of alternative powertrains in agricultural tractors. *World Electr. Veh. J.* **15** (2024).

[13] Kim, Y. S. et al. Development of DEM-MBD coupling model for draft force prediction of an agricultural tractor during plow tillage. *Comput. Electron. Agric.* **202**, 107405. 10.1016/j.compag.2022.107405 (2022).

[14] Kim, B. S. et al. Application of asymmetric gears in a tractor transmission system. *J. Biosyst. Eng.* **50**, 301–309 (2025).

[15] Wen, C. et al. Power density based fatigue load spectrum editing for accelerated durability testing for tractor front axles. *Biosyst. Eng*. **200**, 73–88 (2020).

[16] Kim, W. S. et al. Fatigue life simulation of tractor spiral bevel gear according to major agricultural operations. *Appl. Sci*. **10**, 1–19 (2020).

[17] Kim, W. S. et al. Evaluation of the fatigue life of a tractor’s transmission spiral bevel gear. *J. Terrramech.* **94**, 13–22 (2021).

[18] Nataraj, E., Sarkar, P., Raheman, H. & Upadhyay, G. Embedded digital display and warning system of velocity ratio and wheel slip for tractor operated active tillage implements. *J. Terrramech.* **97**, 35–43 (2021).

[19] Hensh, S., Tewari, V. K. & Upadhyay, G. A novel wireless instrumentation system for measurement of PTO (power take-off) torque requirement during rotary tillage. *Biosyst. Eng*. **212**, 241–251 (2021).

[20] Mocera, F., Somà, A., Martelli, S. & Martini, V. Trends and future perspective of electrification in agricultural tractor-implement applications. *Energies*10.3390/en16186601 (2023).

[21] Kim, N., Yan, Z., Vijayagopal, R., Jung, J. & He, X. Evaluation of advanced powertrain technologies for large agricultural tractors: Insights on energy consumption and life cycle environmental impact. *SAE Int. J. Sustain. Transp. Energy Environ. Policy* **06**, 221–242 (2025).

[22] Kim, N. et al. Development of the power- and usage-based simulator for evaluating off-road mobile machinery energy consumption. SAE Technical Paper. 1–11. 10.4271/2025-01-8595 (2025).

[23] Kim, Y. S. et al. Power transmission efficiency analysis of 42 kW power agricultural tractor according to tillage depth during moldboard plowing. *Agronomy* **10** (2020).

[24] Kim, W. S., Kim, Y. S. & Kim, Y. J. Development of prediction model for axle torque of agricultural tractors. *Trans. ASABE* **63**, 1773–1786 (2020).

[25] Troncon, D. & Alberti, L. Case study of the electrification of a tractor: Electric motor performance requirements and design. *Energies* **13**, 2197. 10.3390/en13092197 (2020).

[26] Mocera, F. & Somà, A. Analysis of a parallel hybrid electric tractor for agricultural applications. *Energies* **13**, 3055. 10.3390/en13123055 (2020).

[27] Deng, X. et al. Research on dynamic analysis and experimental study of the distributed drive electric tractor. *Agriculture* **13**, 40. 10.3390/agriculture13010040 (2022).

[28] Chen, Y. et al. Powertrain parameter matching and optimal design of dual-motor driven electric tractor. *Int. J. Agric. Biol. Eng.* **12**, 33–41. 10.25165/j.ijabe.20191201.3811 (2019).

[29] Lappalainen, T., Petrov, I. & Pyrhönen, J. Agricultural tractor electrical propulsion concept. *Energy Convers. Manag. X* **26** (2025).

[30] Saetti, M., Mattetti, M., Varani, M., Lenzini, N. & Molari, G. On the power demands of accessories on an agricultural tractor. *Biosyst. Eng.* **206**, 109–122 (2021).

[31] Kim, W. S. et al. Influence of tire contact area on the traction performance of a 67-kW agricultural tractor in a paddy field.. *J. ASABE* **65**, 1421–1432. 10.13031/ja.15049 (2022).

[32] Kim, W. S. et al. Traction performance evaluation of a 78-kW-class agricultural tractor using cone index map in a Korean paddy field. *J. Terramech.* **91**, 285–296. 10.1016/j.jterra.2020.09.001 (2020).

[33] Paraforos, D. S., Griepentrog, H. W., Vougioukas, S. G. & Kortenbruck, D. Fatigue life assessment of a four-rotor swather based on rainflow cycle counting. *Biosyst. Eng.* **127**, 1–10. 10.1016/j.biosystemseng.2014.08.003 (2014).

[34] Kim, W. S. et al. Analysis of power requirement of 78-kW class agricultural tractor according to the major field operation. *Trans. Korean Soc. Mech. Eng. A* **43**, 911–922. 10.3795/KSME-A.2019.43.12.911 (2019).

